# Immunomodulation profile of the biosimilar trastuzumab MYL-1401O in a bioequivalence phase I study

**DOI:** 10.1038/s41598-024-61265-2

**Published:** 2024-06-04

**Authors:** R. Audran, H. Chtioui, A. C. Thierry, C. E. Mayor, L. Vallotton, K. Dao, L. E. Rothuizen, A. Maghraoui, E. J. Pennella, F. Brunner-Ferber, T. Buclin, F. Spertini

**Affiliations:** 1 Division of Immunology and Allergy, Centre Hospitalier Universitaire Vaudois (CHUV), University Hospital Lausanne, rue du Bugnon, 1011 Lausanne, Switzerland; 2 Division of Clinical Pharmacology, CHUV- University Hospital Lausanne, Lausanne, Switzerland; 3 Clinical Trial Unit, CHUV - University Hospital Lausanne, Lausanne, Switzerland; 4 Mylan, Canonsburgh, PA USA; 5 Brunner Naga, Pfaeffikon, Switzerland

**Keywords:** Biosimilar, Trastuzumab, mAb, Biomarker, PBMC, Cytokine release assay, Cancer therapy, Phase I trials, Immunology, Immunotherapy, Antibody therapy

## Abstract

The initial Phase-I single centre, single dose, randomized, double-blind, cross-over study was planned to assess the pharmacokinetic and pharmacodynamic bioequivalence of the trastuzumab biosimilar (MYL-1401O) compared to the reference Herceptin^®^. Their respective immunomodulation profile presented in this paper involved healthy males receiving a single infusion of both monoclonals, separated by a washout period. Sixty parameters were assessed in total, including serum cytokines, peripheral mononuclear cell (PBMC) subsets, cell activation and response to recall antigens and mitogen, pre- and post- infusion, as well as a cytokine release assay (CRA) at baseline. Trastuzumab infusion induced a transient and weak peak of serum IL-6 at 6 h, and a modulation of mononuclear cell subset profile and activation level, notably CD16 + cells. Except for CD8 + T cells, there were no significant differences between Herceptin^®^ and MYL-1401O*.* In CRA, PBMC stimulated with MYL-1401O or Herceptin^®^ similarly secreted IL-6, TNF-α, IL-1β, GM-CSF, IFN-γ, and IL-10, but no or low level of IL-2. Interestingly, some observed adverse events correlated with IL-2 and IFN-γ in CRA. MYL-1401O exhibited a very similar immunomodulation profile to Herceptin^®^, strongly supporting its bioequivalence. This approach may thus be included in a proof-of-concept study. CRA may be used as a predictive assay for the evaluation of clinical monoclonals.

## Introduction

Trastuzumab is a humanized IgG1 monoclonal antibody (mAb) which binds to the human epidermal growth factor receptor 2 (HER2). Patients overexpressing this oncoprotein have a poorer prognosis. Trastuzumab was successfully developed by Genentech/Roche for treating patients with metastatic breast cancer overexpressing this oncoprotein (~ 30%). Trastuzumab was registered by the FDA in 1998 and by the EMA in 2000 under the name of Herceptin® with their respective patent protection expiring in June 2019 for USA and in July 2014 for Europe.

Bearing in mind the spectacular medical and commercial success of Herceptin^®^ (worldwide sales in 2018 over US$ 7.5 billion), several companies launched a search for a biosimilar to be compared to reference trastuzumab in the 2010ies. The goal of biosimilars is to improve global access at reduced price for patients. However, the development of a biosimilar is complex, slow, and very expensive, especially during the period when guidelines for biosimilars were being developed at reduced and staggered pace and when the legal status was unclear. Mylan (Canonsburgh, PA, USA) and Biocon (Bangalore, India) entered into such a collaboration agreement to develop MYL-1401O (also known as Hercules and later registered as Ogivri^®^ by FDA in 2017 and by EMA in 2018).

The initial Phase I study selected a crossover design in male volunteers to compare MYL-1410O to the reference Herceptin^®^ for assessing pharmacokinetic (PK) bioavailability, pharmacodynamic (PD) bioavailability based on the antiproliferative activity of serum using a cell-based assay with a breast tumor cell line (BT-474), safety profile and immunogenicity^1^ (Supplementary doc.1). This study also included immunomodulation markers as exploratory objectives for assessing immunological bioavailabilty, and these results are the basis for this article. For this purpose, we selected markers of immunomodulation in relation to the PD of trastuzumab. Its antitumor activity on HER2 over-expressing cancer cells is mainly expressed in two modes, one biological and one implicating the immune system. HER2, or ERBB2, is a membrane receptor of the HER family with an extracellular ligand-binding domain and an intracellular tyrosine kinase domain. Binding of a ligand of the epidermal growth factor (EGF) family to HER receptors induces receptor dimerization, preferentially with HER2, phosphorylation of HER2, followed by intracellular signals essential for proliferation and survival of HER2 positive cancer cells. Binding of trastuzumab on HER2 interferes with EGF-dependent growth factor signaling, inducing G1 cell cycle arrest and apoptosis of the target cell^2^. The second mechanism of action involves antibody-dependent cell-mediated cytotoxicity (ADCC). The exhibition of IgG1 Fcγ fragment of cross-linked trastuzumab on HER2 positive cells will induce the recruitment and the activation of Fc receptor (FcR) expressing innate immune effectors. This triggers phagocytosis of the target cells and the release of inflammatory mediators (including TNF-α, IFN-γ, IL-6) and cytotoxic substances. ADCC can be effected by various FcR (CD16 and CD32) expressing cells, such as natural killer cells (NK), neutrophils and monocytes/macrophages, B cells and dendritic cells^3^. NK cells which express the activating low affinity Fcγ RIIIA (CD16), but no inhibitory Fcγ receptors, are widely believed to be the major mediators of cytotoxic activity^4^. ADCC and overall NK cell activity has been correlated with responses to trastuzumab treatment^5,6^. The therapeutic effect of trastuzumab may depend also on adaptive immunity^7^ but involvement of T and B cells remains unclear.

Therefore, and bearing in mind the presence of HER2 at the surface of normal tissues, we expected to observe modulation of immune cells expressing FcγR. Moreover, infusion of exogenous products could cause inflammation followed by immunosuppression. From a regulatory perspective, it was decided to measure circulating cytokines, and to study various blood cell subsets and their activation status by cytometry. In addition to the tests carried out in direct relation to their mode of action as monoclonals, the analysis of immune modulation response to antigens was also recommended^8–10^. Although there is no evidence in the published literature that T cells can express HER2 (even at a low level)^11,12^, T and B cells may be affected indirectly by the treatment, including through increased levels of circulating cytokines. Finally, when a mAb targets the immune system, Regulatory Authorities recommend performing, pre-clinical in vitro assays based on its mode of action. This should be done in parallel to the analysis of its pharmacodynamics, before injection to humans and in addition to animals experimentation^13^^,^^14^. Although this is obviously not required in the context of a potential biosimilar, we performed a cytokine release assay (CRA) to explore the potential modulation induced ex vivo by trastuzumab complexes on FcγRIII positive cells and its correlation with clinical observations^15–20^. The present paper reports the evaluation of selected dedicated immunological markers, pre and post injection of both monoclonal antibodies, and the attempt to correlate these markers with the observed adverse events.

## Results

### Study volunteers

**Figure 1:**
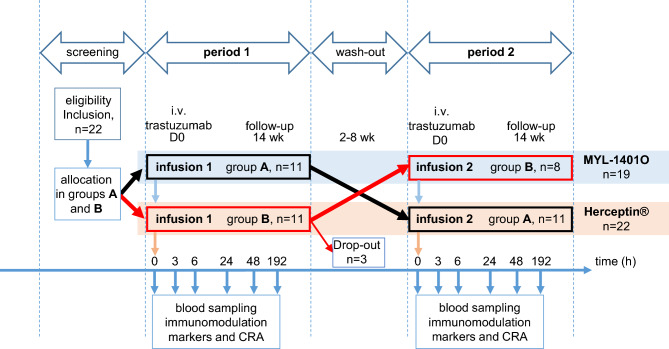
Flow-chart of the study The study was designed as a single centre, single dose, randomized, double-blind, 2-way crossover study in healthy male volunteers. Three drop-outs occurred: two for personal convenience, one for transaminase elevation in period 1.

Twenty-two healthy male volunteers were included in the study and 19 completed both 3 month study periods separated by a 2–8 week washout period. The 3 drop-outs occurred after infusion with Herceptin^®^ after completion of period 1, leading to 22 volunteers infused with Herceptin^®^ and 19 with MYL-1404O (Fig. 1). Demographic characteristics, pharmacokinetics and safety results are summarized in supplementary file.

### Cytokines in serum

**Figure 2:**
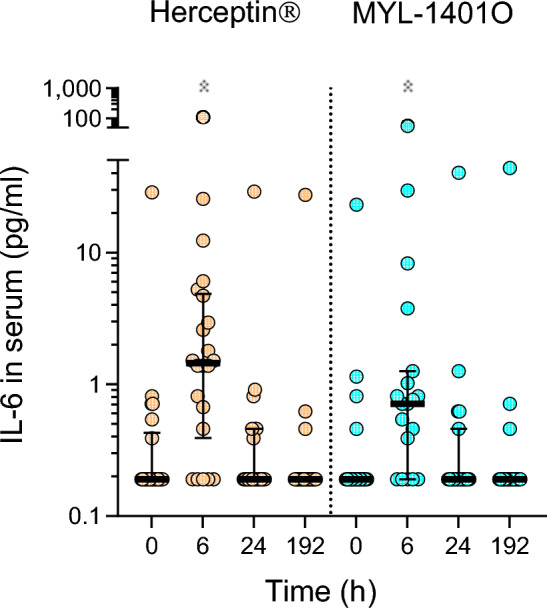
Serum IL-6 concentration The kinetics of IL-6 response after infusion of Herceptin^®^ (n = 22) or MYL-1401O (n = 19) was assessed by Luminex. Results in pg/mL are expressed as geometric mean and CI 95%. Friedman test were performed, Dunns multiple comparison with 0 h are indicated as * when *p* < 0.05.

After administration of MYL-1404O and Herceptin^®^, a peak of IL-6 was observed 6 h post-infusion (p < 0.0001) (Fig. 2). At 24 h, levels of IL-6 returned to baseline. The peak of IL-6 correlated with fever and increased protein C-reactive (CRP) (Supplementary Fig. 1). In contrast, there was no modulation observed for other cytokines IL-1β, IL-2, IL-10, IL-12, IFN-γ, TNF-α, and GM-CSF. No statistically significant differences between MYL-1404O and Herceptin^®^ was observed for any of the 8 circulating cytokines analysed.

### Mononuclear cell subset modulation (frequency and activation state)

**Figure 3:**
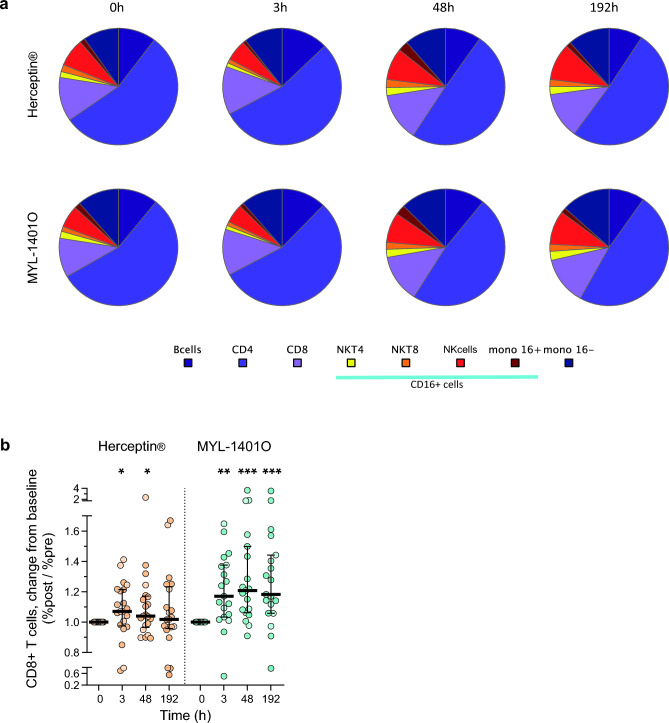
Mononuclear cell populations (**a**). Proportions of mononuclear cell population measured before and 3, 48 and 192 h after infusion of either Herceptin^®^ or MYL-1401O. The median proportion of B cells, CD4 + , CD8 + , NKT CD4 + (NKT4) and NKT CD8 + (NKT8) T cells, NK cells and monocytes expressing CD16 (mono 16 +) or not (mono 16-) is represented by pie charts. (**b**)**.** The ratio of frequencies of CD8 + T cells post-infusion/pre-infusion is represented by medians and quartiles in each treatment group. Wilcoxon signed rank test was performed for comparison with 0 h and significant *p* values indicated as *p < 0.05, **p < 0.01 and ***p < 0.001.

We compared the frequency of the major PBMC subsets pre and post infusion by cytometry. A modulation of cell subsets could be observed and globally, the two treatments induced the same biphasic pattern (Fig. 3a). NK cells decreased at 3 h and were higher at 48 h, and still higher at 192 h (p < 0.0001). NKT cells, CD8 + and CD4 + T cells, followed the same pattern as that of NK cells (p < 0.0001 and < 0.0001, resp.). Monocytes followed more or less the same variations (p = 0.015). On the contrary, B cells peaked at 3 h then decreased (p = 0.0035). The population of T cells, stable at 3 h, decreased at 48 h and 192 h (p = 0.0066). This modulation of T cells was due to a decrease of CD4 + helper T cells (p < 0.0001), the major subset of T cells, although CD8 + T cells increased already as of 3 h post-dose (p = 0.0043). The only difference between treatment arms was detected in the CD8 + T cell response. In fact, MYL-1401O induced a stronger and more prolonged increase of CD8 + T cells compared to Herceptin^®^ (p = 0.013, see Fig. 3b).

Regarding monocytes, 3 subsets with various properties can be distinguished based on their expression of CD14 and CD16 with increasing levels of maturation ranging from classical CD14^+^CD16^-^, anti-inflammatory CD14^+^CD16^+^ to pro-inflammatory CD14^−^CD16^+^ monocytes. Since trastuzumab acts via the interaction of Fc fragment on CD16 + cells, we postulated that these mAbs could affect CD16 + monocytes. Drug administration induced the fluctuation of pro- and anti-inflammatory CD16 + monocytes as already described for NK and NKT cells, with a decrease at 3 h, followed by a marked peak at 48 h and a return to the status before treatment at 192 h (p < 0.0001).

**Figure 4:**
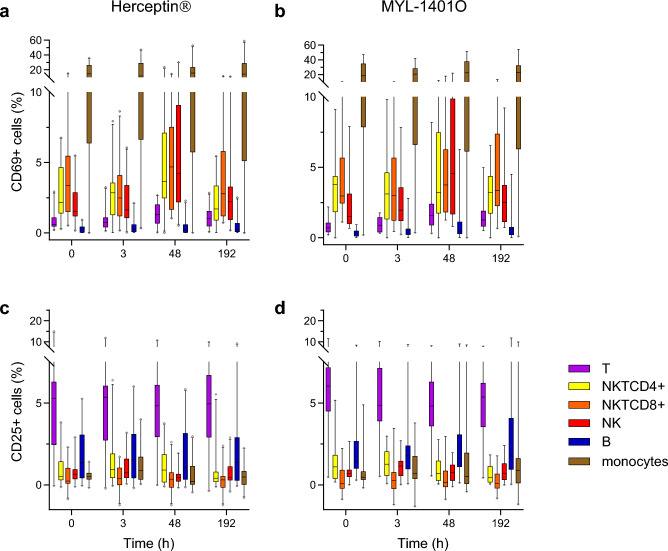
Cell activation marker Frequency of CD69 + (panels A and B) and CD25 + (panels C and D) within various cell subsets were measured pre and post treatment with Herceptin^®^ (**a** and **c**) or MYL-1401O (**b** and **d**). Results are showed as boxplots with median, quartiles and 5% centiles.

Induction of activation markers CD69 and CD25 in response to infusion with trastuzumab was analyzed in each cell subset (Fig. 4). Before treatment, the frequency of CD69-positive cells varied across cells subsets, with monocytes constitutively expressing CD69 and, among lymphocytes, B cells showing the lowest level and NKT cells the highest (Fig. 4a and b). A peak of CD69 + cells was observed at 48 h, for T cells, CD8 + and CD4 + , NK, NKT and B cells (p = 0.0006, 0.012, 0.0012, < 0.0001, 0.0001 and NS). The frequency of CD69 + B cells started to increase at 3 h, while CD8 + NKT cells exhibited instead a decrease in CD69 + cells at 3 h before the peak at 48 h. At 192 h, return to pre-treatment levels was reached for NK and NKT cells. MYL-1401O treatment induced higher levels of CD69 + T cells, particularly CD4 + , than Herceptin^®^ (p = 0.034). On the contrary, Herceptin^®^ induced a higher peak of CD69 + CD4 + NKT cells (p = 0.022).

Basal CD25 expression also varied among cell subsets, with the highest level reached by T cells, particularly CD4 + T cells (Fig. 4c and d). In this population, CD25 expression was slightly downregulated from 3 h and returned to baseline at 192 h (p = 0.013). CD25 + NK cells peaked at 3 h and return to baseline from 48 h (NS). Monocytes and B cells tend to increase their frequencies of CD25 + cells with treatment, particularly after MYL-1401O infusion.

### Stimulation of peripheral blood mononuclear cells with recall antigen and mitogen

**Figure 5:**
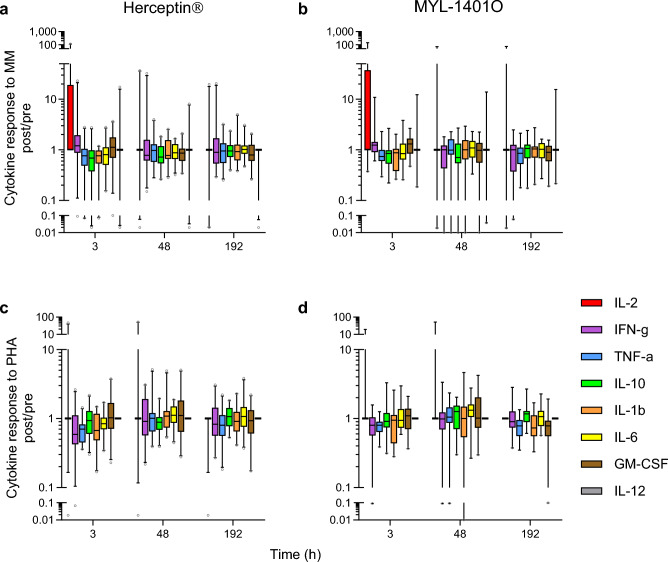
In vitro cytokine response of PBMC to recall antigens and mitogen PBMC were taken at various time points pre and post treatment with Herceptin^®^ (panels **a** and **c**) or MYL-1401O (panels **b** and **d**) and stimulated with a memory mix (MM) (**a** and **b**) or phytohemagglutinin (PHA) (**c** and **d**). Cytokines were measured in 6-day culture supernatants. Results expressed as post/pre-treatment ratio of concentrations are shown as boxplots with median, quartiles and 5% centiles.

PBMCs from MYL-1401O and Herceptin® treated subjects, that were cultured with recall antigens (MM) and mitogen (phytohaemagglutinin, PHA), produced very similar cytokine profiles, depending on stimulation (Fig. 5). There was no statistically significant difference between the two treatments for either stimulus. Stimulated PBMC produced GM-CSF, IFN-γ, IL-1β, TNF-α, IL-2, IL-10, and IL-6, but no or very low levels of IL-12. Unstimulated PBMCs also produced IL-6 and low levels of the other cytokines but with no significant time effect. Globally, in responses to MM, IL-2, GM-CSF and IFN-γ showed an up-regulation 3 h after infusion followed by a decrease at 48 h (p = 0.0021, 0.036 and NS, resp.), although IL-10, IL-1β and TNF-α were down-regulated at 3 h, 48 h and 192 h (p = 0.043, NS and NS, resp.). In response to PHA, TNF-α, IFN-γ and IL-1β were down-regulated at 3 h (p = 0.0002, 0.053 and NS, resp.), and at 192 h for TNF-α, although GM-CSF showed a peak at 48 h (p = 0.0025).

### Baseline in vitro stimulation

**Figure 6:**
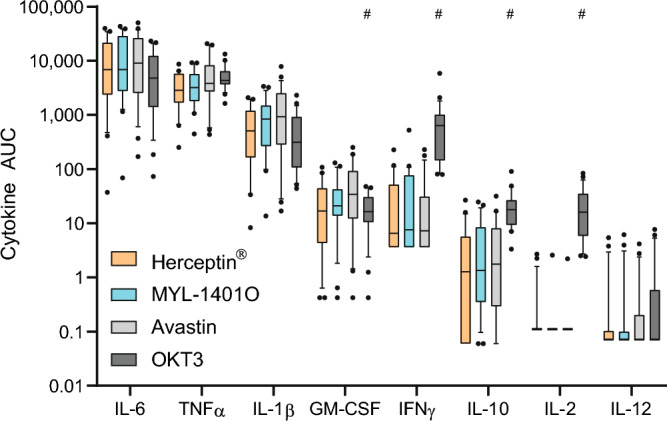
In vitro cytokine response of PBMC to mAbs The cytokine release assay (CRA) was performed with blood taken before the first treatment. PBMC were stimulated with 2 doses of adsorbed monoclonals, Herceptin^®^, MYL-1401O or Avastin^®^ and anti-CD3 (OKT3) as low and high reactogenicity controls. Cytokines were measured in 20 h culture supernatant. Results expressed as area under the curve (AUC) are shown as boxplots with median, quartiles and 5% centiles. Wilcoxon test was used to assessed comparison with Avastin^®^. #p < 0.05.

**Figure 7:**
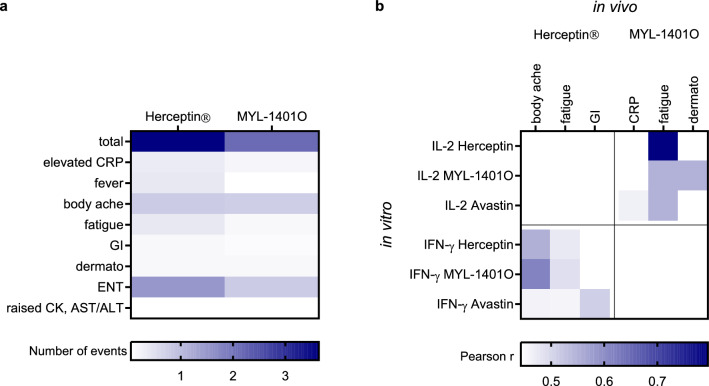
Correlation between CRA and toxicity (**a**). Heat map showing the average number of adverse events (AE) per subject, in total or per category of events, induced by the Herceptin^®^ or MYL-1401O treatments. (**b**). Heat map showing correlation as Pearson *r* between CRA performed on PBMC taken before the first treatment (in vitro) and numbers of AE observed after treatments (in vivo). Cytokines in response to in vitro stimulation of PBMC with Herceptin^®^, MYL-1401O or Avastin^®^ were measured, only IL-2 and IFN-γ gave correlations with a *p* < 0.05 and are presented. GI, gastrointestinal disorder, ENT, ear nose throat disorder.

Pre-treatment PBMCs were stimulated in vitro with mobilized mAbs, MYL-1401O, Herceptin^®^ and controls of low and high reactogenicity, Avastin^®^ and OKT3, respectively (Fig. 6). These mAbs induced all the cytokines studied, except IL-12 which was poorly detected, and with a few exceptions. Importantly, IL-2 was released only with OKT3 stimulation, apart from 4 cases. Compared to Avastin^®^, while OKT3 induced different levels of GM-CSF, IFNg, IL-10, and IL-2, both trastuzumab induced comparable cytokines responses, with no difference between MYL-1401O and Herceptin^®^. The cytokines with the highest response to Herceptin^®^ and MYL-1401O were: IL-6 (upper range 40,000 pg/mL), TNF-α (about 8000 pg/mL), IL-1β (3000 pg/mL), GM-CSF (80 pg/mL), IFN-γ (9 pg/mL), and IL-10 (6–7 pg/mL). There was a dose–response relationship for the in vitro production of IL-6, IL-10, IL-1β, GM-CSF, and TNF-α. Finally, we tried to find correlations between these in vitro cytokine responses and clinical observation after each treatment. The numbers of adverse events (AEs) were low, with a mean [CI 95%] of total AEs per volunteer of 3.64 [2.38; 4.89] and 2.14 [1.22; 3.05] after infusion with Herceptin^®^ and MYL-1401O, respectively (Fig. 7a). Additionally, subjects overexpressing cytokines in CRA were few. Nevertheless, it was possible to find a significant correlation between IL-2 and IFN-γ secretion in response to the different mAbs in vitro and some AEs in response to MYL-1401O and Herceptin^®^, respectively (Fig. 7b). Thus, fatigue, elevated CRP and skin reaction observed in response to MYL-1401O infusion were associated with IL-2 responses in vitro, while body aches, fatigue and gastrointestinal issues in response to Herceptin^®^ infusion were associated with IFN-γ.

## Discussion

First, we have demonstrated in this trial that the ex vivo and in vitro immunomodulatory profiles of both anti-HER2 mAbs (Herceptin^®^ and its biosimilar Myl-1401O) are similar over time and are not significantly different. Second, with the increasing numbers of biological agents presently used in the clinics and their subsequent biosimilars coming on the market, it is of the utmost importance to ensure already in early clinical phases that no unforeseen immunomodulatory effect may occur. One of our objectives in the current study was to determine whether in vitro cell activation may predict in vivo reactogenicity. Despite the limited number of clinical adverse events observed during this pharmacokinetic and -dynamic study, we have obtained preliminary but nonetheless significant insights into the ability of CRA for predicting reactogenicity. Therefore, such an approach may be appealing in the future for evaluation of potential clinical mAbs.

### Ex vivo observations: cytokines in serum and mononuclear cell modulation

No significant differences between MYL-1401O and Herceptin^®^ was observed for any serum cytokine. A transient and weak peak of serum IL-6, one of the major proteins implicated in inflammatory reactions, was observed at 6 h. Although stimulated NK cells and monocytes are able to produce IL-6, IL-1β, and TNF-α, no obvious link could be established between the peak of serum IL-6 and the apparent modulation of these populations or with their activation status in the blood. There is a possibility that the IL-6 originated from another source of cells e.g., the reticuloendothelial system or endothelial cells. However, it was not possible to correlate the levels of cytokines in serum with blood cell populations since sera and cell samples had been collected at different time-points for logistical reasons.

MYL-1401O and Herceptin^®^ exerted similar modulation of the majority of mononuclear cells. It is of interest to stress that all the leucocytes that were modulated expressed CD16. CD16 is FcγRIII, a low affinity and activating receptor for the Fc portion of IgG, particularly IgG1. The triggering of FcγRIII on NK, NKT cells, monocytes, and neutrophils (not studied here), activates these cells and induces an ADCC reaction. ADCC comprises phagocytosis, release of inflammatory mediators, and cytotoxic substances. The capacity of FcγRIII for binding the Fc portion of IgG1 increases when immunoglobulins are multimeric, cross-linked with their specific antigen as immune complexes or at the surface of target cells^21^. This suggests that the subjects who clinically reacted to trastuzumab infusion or induced high level of IL-6 may have had higher levels of HER2 on their non-immune cells than subjects who had no or only mild reactions.

A decrease in CD16 + cells was observed, followed by a peak in CD16 + cells at 48 h. The decrease in CD16 + cells can probably be explained by 2 phenomena. The first one is extravasation from blood to tissues and this has been indeed shown that CD16 + monocytes are more motile than CD16- monocytes and transmigrate rapidly^22^. The second phenomenon is down-regulation of CD16 at the cell surface, a typical event when cells are involved in ADCC^5^.

Since the modulation of cell subsets was analyzed as frequencies of each subset within the PBMC population, the decrease of CD16 + cells could be responsible for the relative increase in B cells observed. However, the possibility that B cells were directly modulated by Herceptin^®^ or MYL-1401O infusion cannot be excluded. The peak of CD16 + cells at 48 h would have counterbalanced the first decrease observed. This second peak was accompanied by an increase in activated cells, i.e*.,* T, B, NKT and NK cells.

### In vitro responses to recall antigen and mitogen

PBMC responses to either recall antigens or mitogen reflects immune functions, PHA activating most blood cells when recall antigens preferentially target T lymphocytes. Both mAb treatments modulated the profile of responses, without significant differences between MYL-1401O and Herceptin^®^. The observed responses to PHA, i.e., increase in GM-CSF at 48 h, decrease in TNF-α at 3 h and return to baseline at 48 h, most probably reflect the modulation of CD16 + cells and of activated cells in vivo. Interestingly, in response to antigen stimulation, we observed, at 3 h, a decrease of IL-10 and an increase of IL-2 and GM-CSF, accompanied in vivo by the decrease of CD25 + T cells. The CD25 + T cell population includes T regulatory cells (T_reg_) cells that produce IL-10, which in turn inhibits production of IL-2, a cascade of events that could be responsible for the observed effect. CD25 is also a late marker of lymphocyte activation while CD69 is an early marker of cell activation. The decrease in CD25 + T cells preceding the peak of CD69 + T cells at 48 h may express an actual loss of T_reg_. However, the mechanism explaining the relationship between the decrease of CD25 + T cells and infusion with MYL-1401O or Herceptin^®^ is not fully elucidated.

### Baseline in vitro stimulation

In vitro stimulation of pre-infusion PBMCs with either MYL-1401O or Herceptin^®^ induced similar cytokine responses. These responses were also similar to that of bevacizumab, Avastin^®^ a mAb of low reactogenicity. Overall, there was no or limited induction of IL-2, which appears to be a good predictor of severe secondary effects such as cytokine storm^16,19^. Altogether, this indicates that MYL-1401O and Herceptin^®^ have a similar profile without safety concern. Fc effector function, such as the direct killing of cancer cells via ADCC or CDC mediated by IgG1, implicates that Fc-mediated side effects may be unavoidable. Indeed, injection of Herceptin^®^ is known to induce mild-to-moderate inflammatory responses characterized by fevers or chills in 40% of patients. Some hypersensensitivity reactions were also observed but only after multiple exposures to trastuzumab (reviewed by Hong 2012^23^). However, in absence of tumor bearing HER2, such as in the current trial, trastuzumab induced limited adverse reactions in healthy volunteers when compared to patients. Although being limited by the number of observations, the analysis of individual responses showed a correlation between observed adverse events and induced IL-2 and IFN-γ in vitro*.* This is an indication that CRA could be used as a general predictive assay for evaluation of future clinical mAbs. Meanwhile, it would be appealing to evaluate the CRA's ability to predict reactogenicity in patients who are starting this type of immunotherapy.

In conclusion, when evaluating pre and post infusion in vivo, ex vivo and i*n vitro* markers, we have demonstrated that both antibodies induce a similar and transient peak of cellular activation, mainly innate, while clinically silent. Immunomodulation initially rated in the protocol as an exploratory approach may thus be included in a proof-of-concept study. Taken together, there is a strong assumption that MYL-1401O is biosimilar to the reference drug Herceptin^®^ for its immunomodulation properties, as also proven using a parallel design in Phase I for PK bioequivalence and for clinical efficacy in Phase III^24,25^.

## Patients and methods

### Study design

This initial study was designed as a single centre, single dose, randomized, double-blind, 2-way crossover study conducted in healthy male volunteers with 3-month study periods separated by a 2–8 week washout period. The study was conducted at University Hospital Lausanne, CHUV, Switzerland. At the beginning of each study period, the volunteers received over 90 min a single 8 mg/kg trastuzumab intravenous infusion of MYL-1401O (Hercules, Ogivri®, Biocon, Bangalore, India as per partnership agreement with Mylan, Canonsburgh, USA) or reference drug Herceptin^®^ (Roche Diagnostics GmbH, Mannheim, Germany, EU formulation) in random sequence. Safety laboratory parameters and immunomodulation markers were monitored throughout the study (Fig. 1). The trial was registered with EudraCT (2011-001406).

### Blinding

The solution of study drug, Hercules or Herceptin^®^ administered to each subject by clinical pharmacologists were prepared by pharmacists, under secrecy agreement, using the allocation schedule provided by an independent statistician. All the analyses described in this paper and in supplement were performed blind by immunologists and other study partners. Decoding could only be performed after the database was blocked.

### Blood samples

For immunomodulation assessment, peripheral blood mononuclear cells (PBMC) from blood taken prior to each injection, at 3 h, 48 h and 192 h post-infusion were separated on a density gradient (Ficoll-Paque Plus, GE Healthcare Biosciences, S), resuspended in 90% FCS 10% DMSO and stored in liquid nitrogen until analysis. Sera were taken before injection, at 6 h, 24 h and 192 h post-infusion and were stored at − 80 °C until analysis.

### Cell stimulation

We analysed the kinetics of response in vitro to recall antigens and to mitogen which should reflect the variation of cell populations and/or their activation status, as previously described^26^. After thawing, PBMC were distributed in 96- round-bottom well plates (2 × 10^5^ cells/well) in 0.2 mL RPMIc: RPMI (Sigma Chemical Co, St. Louis, MO, USA) supplemented with glutamine (Sigma), 100 IU/mL penicillin–streptomycin, 100 µM non-essential aminoacids, kanamycin, 2 mM sodium pyruvate (Invitrogen), 8% human AB serum (AB, Blutspendedienst SRK Bern AG, CH-Bern). Cells were stimulated for 6 days in triplicates with a memory mix (MM), a cocktail of PPD (tuberculin RT50, SSI, Copenhagen, Denmark, 1 µg/mL), tetanus toxoid (TT, Pasteur Mérieux, Lyon, France, 2 µg/mL) and Candida mannan (Candida albicans, NIBSC, Hertfordshire, UK, 0.4%), with phytohaemagglutinin (PHA, Sigma, 2 µg/mL) or unstimulated as controls. For the CRA, we stimulated PBMC taken before the first infusion with mobilized mAbs. These antibodies include trastuzumab (anti-HER2, IgG1, Herceptin^®^, Roche, Basel, Switzerland and MYL-1401O, Biocon, Bangalore, India as per partnership agreement with Mylan, Canonsburgh, USA), bevacizumab (anti-VEGF, IgG1, Avastin^®^, Roche, Basel, Switzerland) and muromonab-CD3 (anti-CD3, murine IgG2a, Orthoclone^®^3, OKT3, Janssen-Cilag, Baar, Switzerland). Escalating doses from 0.01 to 10 μg of antibodies in 50 µL of phosphate buffer saline (PBS) were air-dried onto 96 well polypropylene (PP) microtitre plates (Corning, NY) overnight at room temperature, followed by two washes with PBS, as described^17^. PBMC (2 × 10^5^ cells/well) in 0.25 mL RPMIc were cultivated for 20 h in duplicates with these mobilized antibodies, or not stimulated. Cell supernatants were collected at the end of cultures (6 days or 20 h) and stored at− 80 °C until cytokine analysis.

### Cytokine release profile

IL-1β, IL-2, IL-6, IL-10, IL-12, IFN-γ, TNF-α, and GM-CSF were measured in culture supernatants and in sera by multiplexed particle-based flow cytometric assays (Bio-plex Pro^™^, Human Cytokine Standard group I, Bio-Rad Laboratories, Hercules, CA, USA) according to manufacturer’s instructions. Acquisition of mean fluorescence intensity (MFI) was performed using a Luminex 100 (Thermo Fisher Scientific). As IL-6 in culture supernatants frequently reached the highest limit of detection, it was further analyzed on serial dilutions of samples, using an ELISA kit (BD OptEIA^™^ Set human IL6, BD Biosciences). Optical densities (OD) were acquired using a microplate photometer (Opsys MR^™^, Dynex, Worthing, UK). Raw data, MFI and OD, were analyzed in Excel and converted to concentrations (pg/mL) using standard curves. Area under the curve (AUC) were calculated for dose-responses using GraphPad prism.

### Ex vivo analysis of leukocyte subset modulation (frequency and activation state)

PBMC from the same individual, collected at various time-points were thawed and analyzed in parallel. Cells were stained with a cocktail of immune-fluorescent antibodies: CD3-PerCP Cy5.5, SK7, CD8-Pacific Blue, RPA-T8, CD56-PE, B159, CD32-APC, FLI8.26, CD14-APC-Cy7, MOP1, CD25-PE-Cy7, MA251, all from BD Pharmingen, CD16-A700, 3G8 and CD69-FITC, FN50 from Biolegend, CD19-ECD, J3-119, Beckman Coulter and Vivid AmCyan (Thermo Fisher Scientific) for dead cell exclusion. Additionally, pre-treatment PBMC were labeled with the cocktail containing isotype controls IgG MOPC21 in FITC (Biolegend) and PE-Cy7 (BD) in place of activation markers. Cells were collected on an LSR-II and frequencies of the various cell subsets and their activation status upon expression of CD69 and CD25 analysed on FACS-DIVA. Live cells subsets were distinguished according to their morphology (SSC, FCS) and the expression of their cell surface markers: T lymphocytes: SSC^lo^ CD3^+^ (CD19 CD14 CD32)^-^, CD56^+^ (NKT cells) and CD56^-^ (CD8^+^ and CD4^+^ T cells), NK cells: SSC^lo^ CD16^+/-^ CD56^+/−^ (CD3 CD19 CD32 CD14)^-^, B lymphocytes: SSC^lo^ CD19^+^ (CD3 CD56 CD14)^-^, Monocytes: SSC^hi^ CD32^+^ (CD19 CD56 CD3)^-^, divided in classical CD14^+^CD16^-^, intermediate CD14^+^CD16^+^ with an anti-inflammatory profile, and non-classical pro-inflammatory CD14^-^CD16^+^ subsets. The gating strategy is illustrated in supplementary Fig. 2a^27^. For each volunteer, cells collected before the first infusion and stained with the isotype corresponding to anti-CD25 and CD69 were used to set thresholds (0.1–0.5%) for each subset studied. These thresholds were used to assess negative and positive (activated) populations for these markers in fully stained samples (Supplementary Fig. 2b). Results were expressed as frequencies of cell subsets over PBMC and % of activated cells in each subset.

### Statistical analysis

Statistical calculations were performed using the Stata v12.1 software on 19 subjects completing the study (as per protocol set). A repeated measures analysis of variance (ANOVA) was used to model the results after logarithmic transformation when appropriate, including sequence, treatment and sampling time and subject. The effect of time was evaluated as the main criterion for the existence of a global response to both trastuzumab formulations. A difference between MYL-1401O and Herceptin^®^ was evaluated by considering the significance of the treatment effect, and treatment × time interaction. Integrated effect and maximal response analysis was also performed when appropriate. Illustrated in figures, analysis of changes over time or comparison with a control were assessed on 22 subjects using Wilcoxon signed rank tests, Friedman with Dunn’s post-tests or 2way ANOVA with Dunnett’s post-tests when appropriate using GraphPad prism v8.0.1 or SPICE 5^28^, respectively.

### Ethics approval

This study was performed in line with the principles of the Declaration of Helsinki and was approved by the competent ethics committee (Commission cantonale d’éthique de la recherche sur l’être humain du canton de Vaud, reference number CER-VD 138/11) and regulatory authority (Swissmedic, reference number 2011DR1104).

### Consent to participate

Written informed consent was obtained from all individual participants included in the study.

### Supplementary Information

Supplementary Information.

## Data Availability

Data are available upon request from François Spertini.

## Abbreviations

ADCC: Antibody-dependent cell-mediated cytotoxicity
AE: Adverse event
CRA: Cytokine release assay
EGF: Epidermal growth factor
FcR: Fc receptor
GM-CSF: Granulocyte–macrophage colony-stimulating factor
HER2: Human epidermal growth factor receptor-2, ERB2, Erb-B2 receptor tyrosine kinase 2
IFN: Interferon
IL: Interleukin
mAb: Monoclonal antibody
PBMC: Peripheral blood mononuclear cells
PD: Pharmacodynamic
PHA: Phytohaemagglutinin
PK: Pharmacokinetic
TNF: Tumor necrosis factor

## Code availability

The classic ANOVA function of the Stata v12.1 software was used for the analysis of variance. Codes are available upon request from François Spertini.

**Table:**

Antibodies	clone	RRID
CD3-PerCP Cy5.5	SK7	RRID: AB_2868620
CD8-Pacific Blue	RPA-T8	RRID: AB_397058
CD56-PE	B159	RRID: AB_395906
CD32-APC	FLI8.26	RRID: AB_398665
CD14-APC-Cy7, MOP1		RRID: AB_1645725
CD25-PE-Cy7	MA251	RRID: AB_396847
CD16-A700	3G8	RRID: AB_2278418
CD69-FITC	FN50	RRID: AB_314839
CD19-ECD	J3-119	RRID: AB_2940901
mIgG1-PE-Cy7	MOPC-21	RRID: AB_396914
mIgG1-FITC	MOPC-21	RRID: AB_326429
Software		
GraphPad Prism,		RRID:SCR_002798
BD FACSDiva Software		RRID:SCR_001456
SPICE		RRID:SCR_016603
Microsoft Excel		RRID:SCR_016137
Stata		RRID:SCR_012763
