# Supplementary material for: Immunomodulation profile of the biosimilar trastuzumab MYL-1401O in a bioequivalence phase I study
Source: Sci Rep. 2024 Jun 4;14:12872. doi: 10.1038/s41598-024-61265-2 (PMC11150559; doi:10.1038/s41598-024-61265-2)
Supplement: Supplementary file 1 — Supplementary Information. [file 41598_2024_61265_MOESM1_ESM.pdf]

Immunomodulation profile of the biosimilar trastuzumab MYL-1401O in a bioequivalence phase I study

Authors:

R. Audran, H. Chtioui, A.C. Thierry, C.E. Mayor, L. Vallotton, K. Dao, L. E. Rothuizen, A. Maghraoui, E.J. Pennella, F. Brunner-Ferber, T. Buclin, and F. Spertini

Supplementary documents

Contents

Supplementary Figure 1 2
Supplementary Figure 2 3
Supplementary document, Chtioui H. (2015) 5

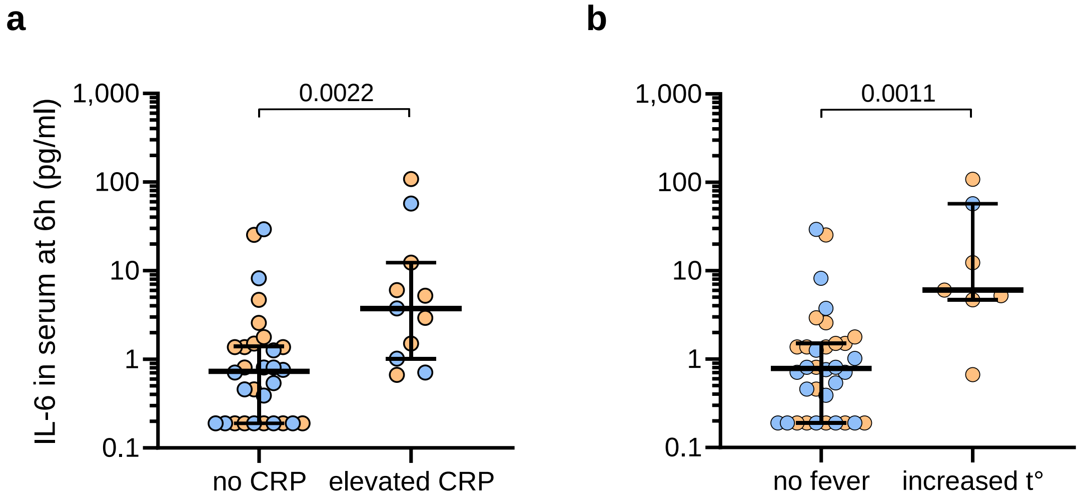

Supplementary figure 1. Correlation between serum IL-6 and CRP level or fever.
The panels show the concentration of IL-6 at 6h in the sera of volunteers showing or not an increase of their levels of protein C-reactive (CRP, panel a) and showing or not fever (panel b), after infusion of Herceptin® (n=22, ●) or MYL-1401O (n=19, ●). Bars indicates medians and quartiles. p values of Mann-Whitney tests are indicated.

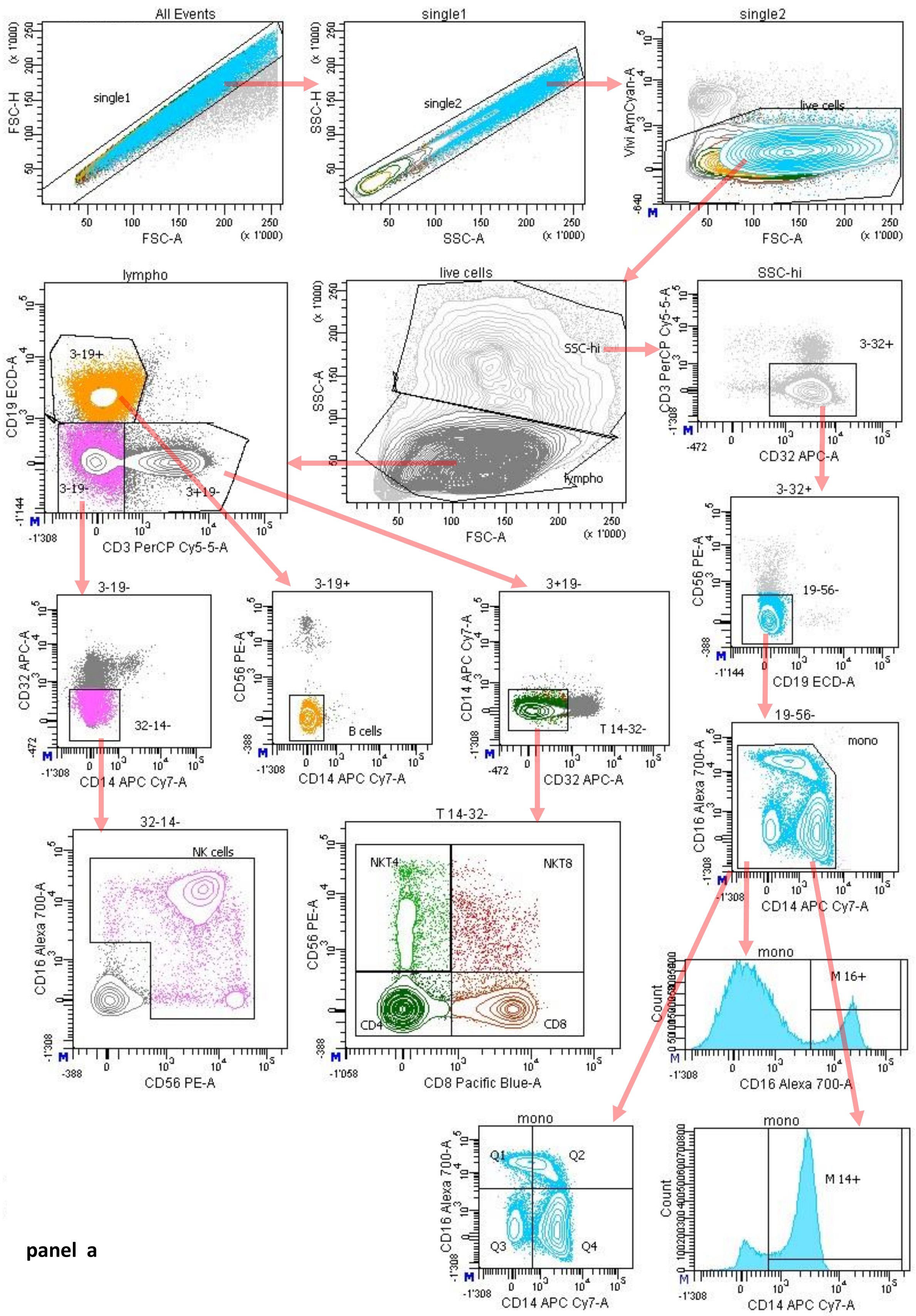
All Events
single1
FSC-H
FSC-A
single2
SSC-H
SSC-A
live cells
Vivi AmCyan-A
lympho
3-19+
3-19-
3+19-
CD19 ECD-A
CD3 PerCP Cy5-5-A
SSC-hi
3-32+
CD3 PerCP Cy5-5-A
CD32 APC-A
19-56-
CD56 PE-A
CD19 ECD-A
32-14-
CD32 APC-A
CD14 APC Cy7-A
B cells
CD56 PE-A
T 14-32-
CD14 APC Cy7-A
CD32 APC-A
mono
CD16 Alexa 700-A
NK cells
CD56 PE-A
NKT4
NKT8
CD4
CD8
CD8 Pacific Blue-A
M 16+
Count
Q1
Q2
Q3
Q4
M 14+

panel a

panel b

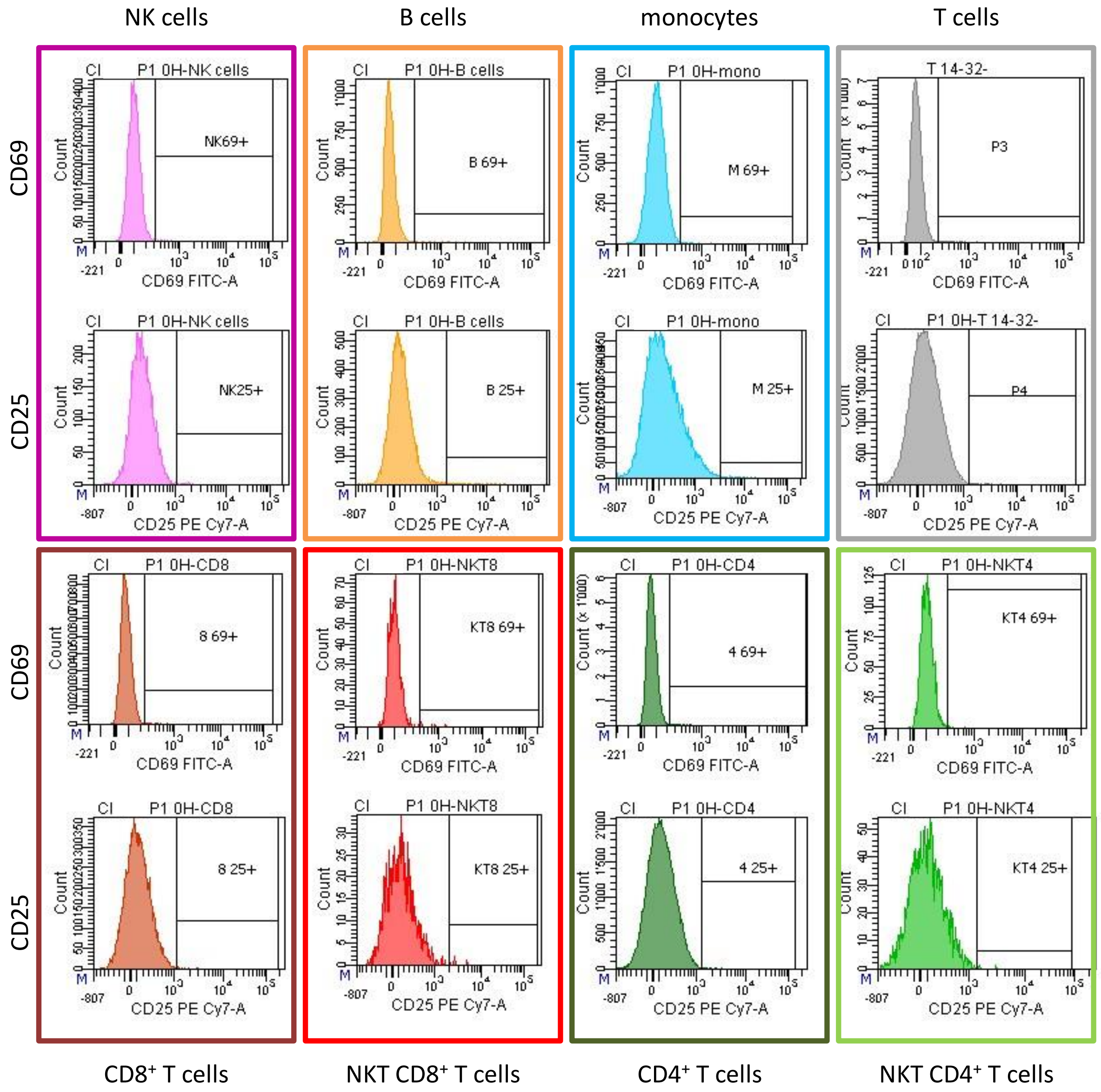

Supplementary figure 2. Gating strategy for the analysis of leukocyte subset modulation.
panel a: gating strategy to select different cell subsets from PBMC. **panel b:** Activation markers, CD69 and CD25, in various subsets of cells. The vertical cursors demarcate positive and negative cells. For each subset of cells, the position of cursors is determined based on isotype control (CI) at t=0h. These positions are used for the following analyses.

BPS 2015, Poster N°: 0370

A Bioequivalence Study for Hercules, a Biosimilar Traztuzumab Candidate in development

H. Chtioui[1], L. Vallotton[1], R. Audran[2], K. Dao[1], L.E. Rothuizen[1], U. Winterfeld[1], M. Appenzeller[1], A. Maghraoui[1], R. Bamford[3], A. Battle[3], E. Pennella[4], F. Brunner-Ferber[5], F. Spertini[2], T. Buclin[1]

[1]Division of Clinical Pharmacology – [2]Division of Immunology and Allergy - CHUV- University Hospital Lausanne,

[3]Covance Harrogate UK , [4] Mylan- Canonsburgh -PA -USA , [5] Brunner-Naga – Pfaeffikon – Switzerland

Introduction

Trastuzumab, marketed as Herceptin®, is a humanized IgG1 monoclonal antibody which binds the human epidermal growth factor receptor 2 (HER2). It is effective against breast cancer overexpressing HER2. We here report the results of a Phase 1 study designed to investigate bioequivalence between Hercules and Herceptin® in healthy volunteers. The assessment of biosimilarity included not only pharmacokinetic (PK) but also pharmacodynamic (PD) equivalence.

Objectives

Primary objective:

- Ascertain **PK bioequivalence** of Hercules and Herceptin® after 8 mg/kg as a single intravenous dose infused over 90 min in healthy males.
  (Bioequivalence is established if 90% confidence interval of the mean ratio of Hercules to Herceptin® - F_{rel} - meets the standard bioequivalence criterion of 80-125% for AUC_{0-inf} and C_{max}).

Secondary objectives:

- Assess comparative systemic safety and tolerability including local tolerance,
- Evaluate immunogenicity and anti-drug antibody (ADA) formation.

Exploratory objectives:

- Compare anti-proliferative activity of serum samples on SKBR3, a cell line overexpressing HER2.
- Investigate PD parameters such as *ex vivo* immunomodulation in PBMCs, cytokine production, apoptosis.

Methods

- Single center, single dose, 2-period, randomized, double-blind, crossover study in healthy males.
- Serum samples for PK, safety and PD markers (proliferation inhibition, immunomodulation) followed over 14 weeks.
- Raw concentrations determined by ELISA with a validated method (Covance Lab.- Harrogate, UK)
- Anti-proliferative activity of serum assessed using a cell-based assay with a breast tumor cell line (BT-474) overexpressing HER2 : proliferation quantified by measuring ATP, expressed in relative luminescence unit (RLU); results translated into relative proliferation inhibition index (iPI).
- Detailed immunological workout (not shown here).

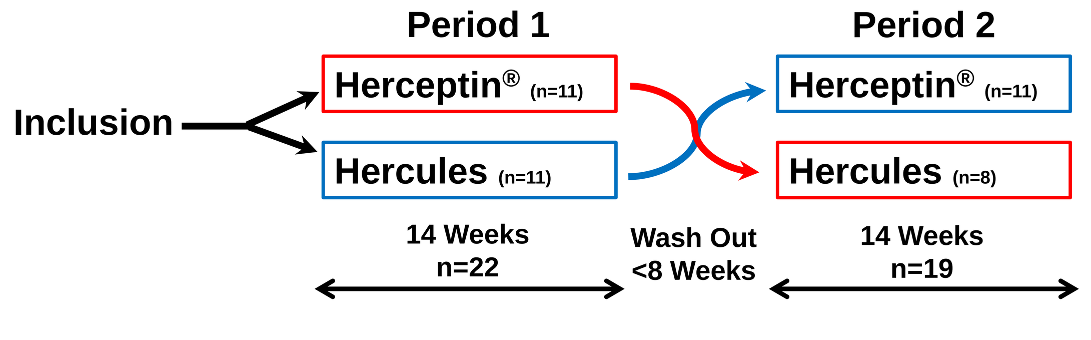

Results

Study subjects :

22 included, 19 analyzed, 3 drop-outs
(2 for personal convenience, 1 for transaminase elevation in period 1).

Table 1. Main demographic charachteristics:

Characteristic	N=19
Age, mean ± SD (years)	27.4 ± 7.0
Weight, mean ± SD (kg)	78.1 ± 13.7
BMI, mean ± SD (kg/m²)	23.6 ± 3.2

BMI = Body mass index; N = Number of subjects; SD = Standard deviation.

Pharmacokinetics :

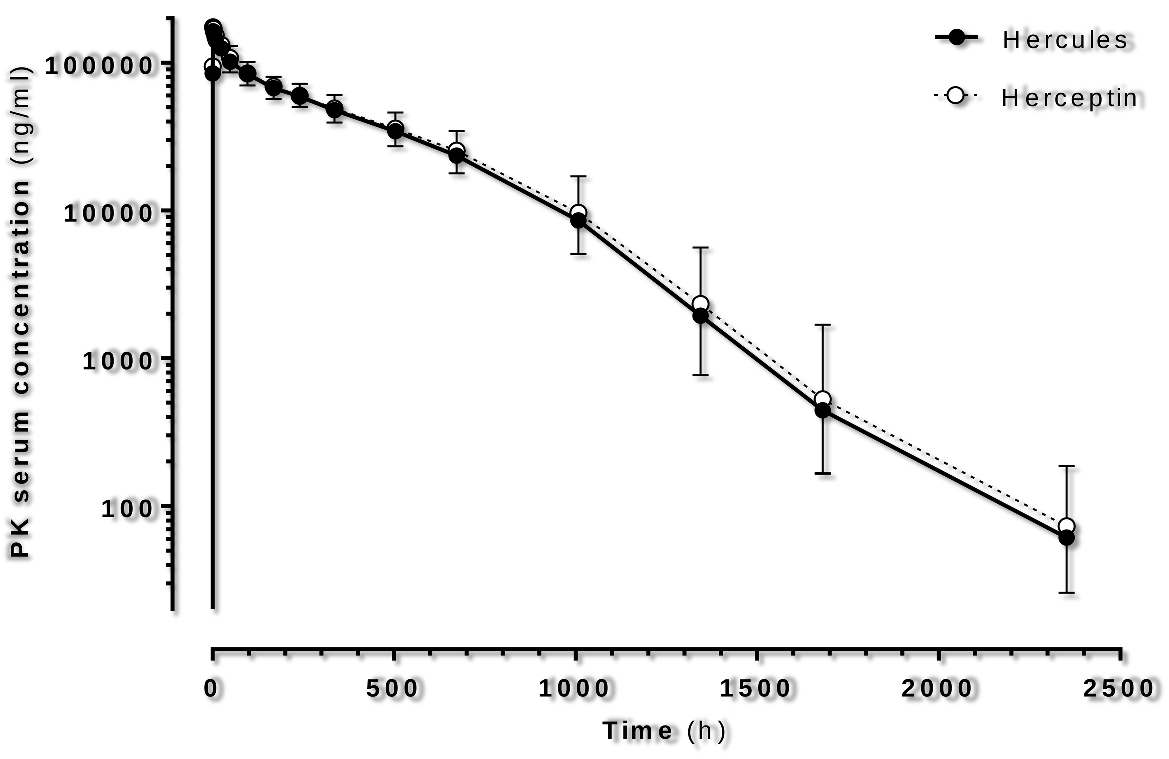

Figure 1. Geometric mean serum concentrations ± geoSD of Hercules and Herceptin®

Table 2. Summary of Pharmacokinetic parameters:

Parameter	Units	Hercules GeoMean (GeoCV%)	Herceptin® GeoMean (GeoCV%)	F_{rel} Point Estimate (90% CI)
C_{max} normalized[a]	µg/mL	165 (15.7)	178 (15.6)	0.922 (0.876; 0.970)
$AUC_{0-\infty}$ normalized[a]	µg.h/mL	45486 (22.7)	48350 (28.5)	0.937 (0.887; 0.989)
T_{max} (median [range])	h	1.5 [1.4-9.0]	1.5 [1.3-9.0]	-
$T_{1/2}$	Day	6.94 (22.6)	7.02 (26.3)	0.988 (0.943; 1.035)
CL[b]	L/day	0.296 (22.7)	0.278 (28.5)	1.068 (1.011; 1.127)

[a] Normalized to 8.0 mg/kg dose; [b] Clearance normalized to 70 kg body weight; GeoCV% = Geometric Coefficient of Variation

Inhibition of Proliferation:

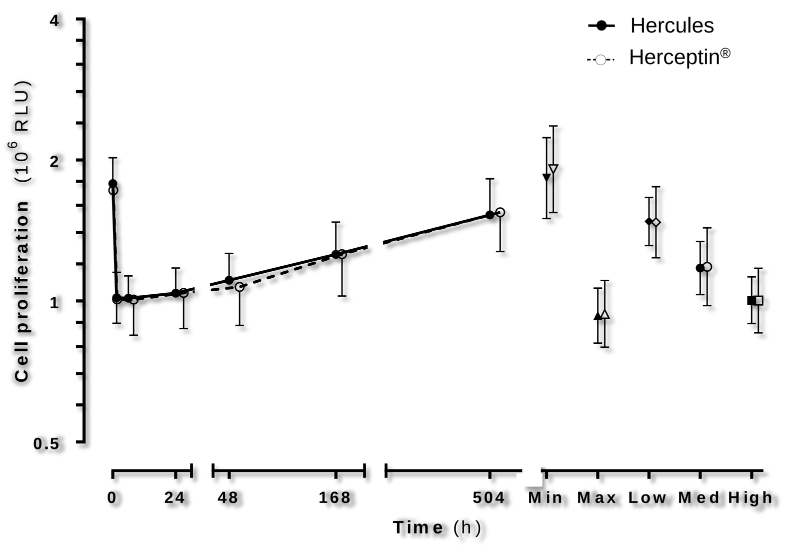

Figure 2. Activity of serum trastuzumab on HER2+ cell proliferation (CP)

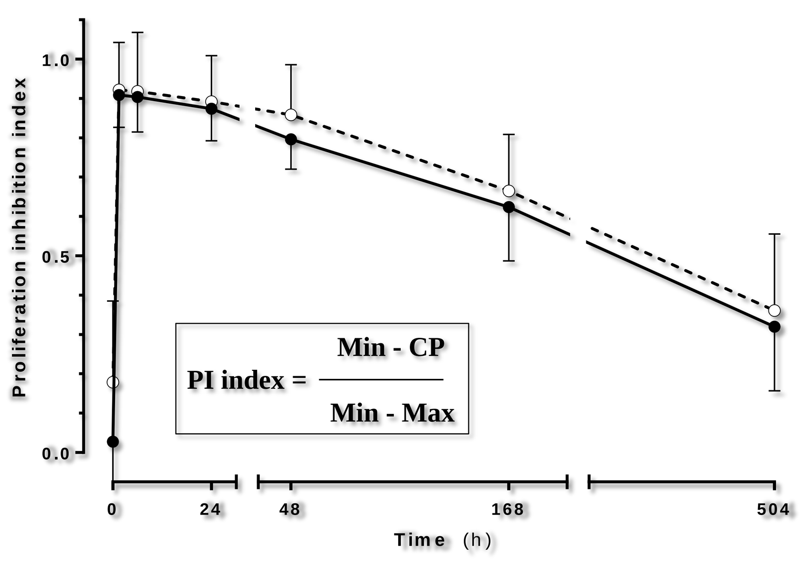

Figure 3. Average proliferation inhibition index (iPI) values (mean ± SD)

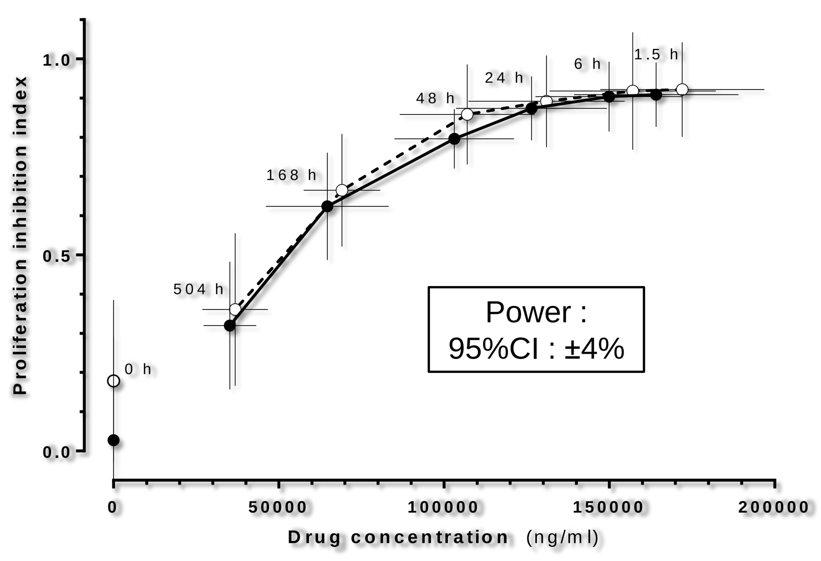

Figure 4. Relationship between average values of trastuzumab concentration and iPI at each time point (mean ± SD)

Safety :

There were no serious adverse events in either groups

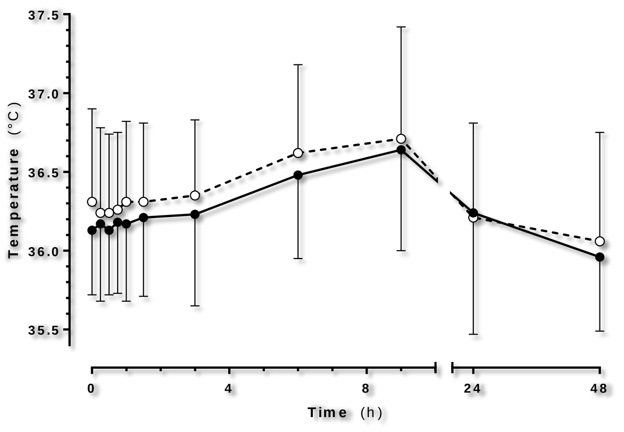

Figure 5. Body temperature

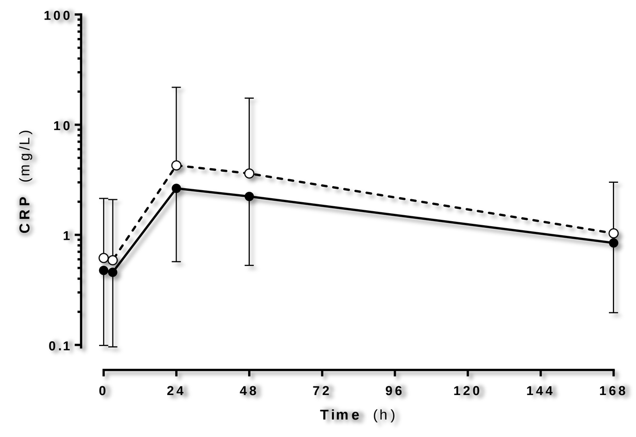

Figure 6. C-Reactive Protein (CRP)

Table 3. Most frequently reported (n ≥ 2) treatment emergent adverse events

System organ class/ Preferred term	No. (%) of subjects Hercules (N=19)	Herceptin® (N=22)
**All**	**16 (84.2)**	**21 (95.5)**
Headache	9 (47.4)	10 (45.5)
Nasopharyngitis	5 (26.3)	12 (54.5)
CRP increase	4 (21.2)	7 (31.8)
Rhinitis	3 (15.8)	8 (36.4)
Fatigue	3 (15.8)	3 (13.6)
Aphthous stomatitis	2 (10.5)	1 (4.5)
Arthralgia	2 (10.5)	0
Rash macular	2 (10.5)	0
Pyrexia	1 (5.3)	4 (18.2)
Pharyngitis	1 (5.3)	2 (9.1)
Influenza like illness	0	3 (13.6)

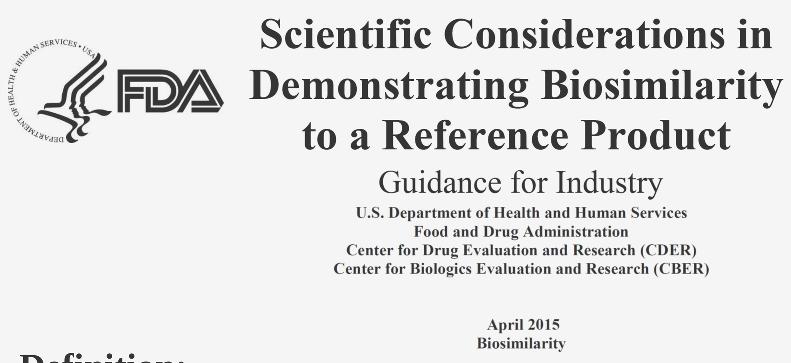

Definition:

Biosimilar or biosimilarity means that "the biological product is highly similar to the reference product notwithstanding minor differences in clinically inactive components," and that "there are no clinically meaningful differences between the biological product and the reference product in terms of the safety, purity, and potency of the product."

1. Human Pharmacology Data

…A **human PK study** that demonstrates similar exposure (e.g., serum concentration over time) for the proposed product and the reference product may **provide support for a demonstration of biosimilarity**.

…A **human PD study** that demonstrates a similar effect on a relevant PD measure(s) related to effectiveness or specific safety concerns (except for immunogenicity, which is evaluated separately) **represents even stronger support** for a biosimilarity determination.

…In certain cases, establishing a **similar clinical PK, PD, and immunogenicity profile** may provide sufficient clinical data to support a conclusion that there are no clinically meaningful differences between the two products.

…**PK and PD parameters are generally more sensitive than clinical efficacy endpoints** in assessing the similarity of two products.

2. Clinical Immunogenicity Assessment

…**FDA encourages** that, where feasible, sponsors **collect immunogenicity data in any clinical study, including human PK or PD studies**.

Conclusions

- Our results confirm pharmacokinetic bioequivalence for Hercules vs. Herceptin®.
- Antiproliferative activity time curves and concentration-effect relationships are similar as well.
- Safety profiles are comparable in healthy volunteers.
- This phase 1 study provides strong support for biosimilarity.
